# Restoration of Motion-Blurred Image Based on Border Deformation Detection: A Traffic Sign Restoration Model

**DOI:** 10.1371/journal.pone.0120885

**Published:** 2015-04-07

**Authors:** Yiliang Zeng, Jinhui Lan, Bin Ran, Qi Wang, Jing Gao

**Affiliations:** 1 Department of Instrument Science and Technology, School of Automation and Electrical Engineering, University of Science and Technology Beijing, Beijing, P.R. China; 2 School of Transportation, Southeast University, Nanjing, Jiangsu Province, P.R. China; University of Chinese Academy of Sciences, CHINA

## Abstract

Due to the rapid development of motor vehicle Driver Assistance Systems (DAS), the safety problems associated with automatic driving have become a hot issue in Intelligent Transportation. The traffic sign is one of the most important tools used to reinforce traffic rules. However, traffic sign image degradation based on computer vision is unavoidable during the vehicle movement process. In order to quickly and accurately recognize traffic signs in motion-blurred images in DAS, a new image restoration algorithm based on border deformation detection in the spatial domain is proposed in this paper. The border of a traffic sign is extracted using color information, and then the width of the border is measured in all directions. According to the width measured and the corresponding direction, both the motion direction and scale of the image can be confirmed, and this information can be used to restore the motion-blurred image. Finally, a gray mean grads (GMG) ratio is presented to evaluate the image restoration quality. Compared to the traditional restoration approach which is based on the blind deconvolution method and Lucy-Richardson method, our method can greatly restore motion blurred images and improve the correct recognition rate. Our experiments show that the proposed method is able to restore traffic sign information accurately and efficiently.

## Introduction

With the development of urbanization and the popularization of the automobile, problems associated with road traffic congestion, frequent traffic accidents, and the low efficiency level of road transport has become increasingly more serious [1]. In order to alleviate these problems, a Driver Assistance System (DAS) was designed to help or even substitute human drivers to enhance the safety of driving [2,3]. This system films the road information in its natural scene using a camera that is mounted inside the vehicle, and this information is subsequently processed in real time using a relevant circuit system. Then, the system provides information, such as warnings and tips, to the driver. This can greatly reduce driving risks and enhance road traffic and the driver's personal safety. Strictly complying with the traffic rules can improve vehicle safety performance, and it can also effectively reduce traffic accidents. A variety of important traffic signs placed on the road by the traffic department communicates and supports road traffic rules for the driver [4]. Traffic signs are designed to help drivers with piloting tasks while providing information, such as the maximum or minimum speed allowed, the shape of the road, and any forbidden maneuvers. Therefore, recognition of traffic signs is one of the important tasks of the DAS in Intelligent Transportation.

The fast detection and accurate identification of traffic signs hold great significance for automatic vehicles. The ability to project a sharp image is one of the preconditions to correctly recognizing a traffic sign. However, the relative motion between the camera and the natural scene during the exposure time usually causes motion-blurred images, which will severely affect the image's visual quality. It is a challenge to quickly and accurately identify traffic signs in motion-blurred images. There are two main approaches used to solve this problem. First, by improving the performance index of the camera, we can avoid the motion blur from a hardware perspective of image processing. However, there are bottlenecks in technology that affect the camera's performance. The second way is to enhance and restore the motion-blurred images by means of a motion-blurred image restoration algorithm. There are also additional things we can do in this field to enhance image quality.

In recent years, many scholars across the world have performed extensive research on the algorithm to resolve image blur degradation. An early and popular method tried to estimate the parameters of motion blur by inspecting the zero pattern of the blurred image in the spectral domain, as there were regular patterns of zeros, depending on the blur scale (*L*) and the blur direction (*θ*). This method was usually used in uniform velocity or close to it, but it failed to deal with images that featured noise. With the advancement of research, some new or modified algorithms have been proposed to improve recognition precision. Ji and Liu [5] and Sun [6] utilized image gradients to enhance this periodic pattern. In addition to Radon transformation, image gradients provided a robust algorithm to catch this periodic pattern, even in cases of noisy images. Ko and Kim [7] extracted the main ripple components from the log spectrum and then determined the angle of motion by measuring the direction of the main ripple along the x-axis. The authors utilized the width of the main ripple to estimate the length. A unified framework, which used bilateral filtering and gradient attenuation based on the Richardson-Lucy algorithm [8,9], was proposed to by Yang et al solve the notorious ringing problem for both blur kernel estimation and non-blind image deconvolution from single blurred image [10]. In addition, Kotera et al. [11] presented a straightforward maximum a posteriori method, combined with sparse priors and an efficient numerical method, which based on blind deconvolution, to recover the unknown features of a single observed blurred image. In another study, Zhang et al. [12] quickly estimated the extent of image blur via wavelet analysis. Furthermore, Deahpande and Patnail [13] used improved dual Fourier spectrum to accurately estimate specific parameters, and they also prevented the ringing effect from occurring at the image boundaries. Wang et al. [14] took both the Hough transform and error-parameters to estimate the blur parameters for linear motion blur, and they also applied curve-fitting and polar transformations to estimate the parameters used to rotate the motion blur. Singh, Tiwary and Kim [15] used an exponent for the correction ratio of Richardson-Lucy (RL) method and accelerated RL method adaptively. Paclik et al. [16] used a correlation function between the logarithm power spectrum, as well as a detecting function to restore the image, and their results also seemed promising in the presence of noise.

Nowadays, the recognition of traffic sign has also made great progress. The Hough transformation and a multi-frames validation method were used by Gonzalez and Garrido [17]. A system based on deformable models was studied, and it was immune to lighting changes, occlusions and other forms of image variance and noise [2]. Support vector machines (SVMs) were utilized to detect and recognize traffic signs by Bascon et al [18]. In addition, Khan et al. [19] proposed a method based on image segmentation and joint transform correlation, which also integrated shape analysis. Barnes et al. [20] also presented the radial symmetry detector to detect speed signs in real time.

All of these algorithms that have been used to restore motion-blurred images took place in the frequency space. Moreover, traffic sign recognition algorithms tend to focus on traffic sign detection and recognition. They do little to deal with traffic signs in blurred images. In order to solve this problem, a new algorithm based on traffic sign border extraction is proposed as a method that can be used to restore motion-blurred images in the spatial domain. The border of the traffic sign is extracted using the image’s color information, and then the width of the border can be measured in all directions. According to the width measured and the corresponding direction analyzed, the motion direction and scale of the image can be confirmed, and then it can be used to restore the motion-blurred image. This method has a lower computational cost and better performance. Meanwhile, the restored image ensures accurate and reliable detection of the traffic sign.

The remainder of this paper is organized as follows: Section II presents the generation of the motion-blurred image and the restoration principle of the motion-blurred image. In Section III, the parameters extraction model, which is based on border deformation detection, is given. Border parameter extraction algorithms are discussed in detail in Section IV. Experimental results are presented in Section V. Finally, a conclusion is presented in Section VI.

## Methods

All of the experimental images in the paper are from the German Traffic Sign Recognition Benchmark (GTSRB) [21]. Physical traffic sign instances are unique within the dataset. There are more than 40 classes and more than 50000 images in total. In addition, the GTSRB dataset is free to use.

### 2.1 Restoration principle of motion-blurred images

**Fig 1:**
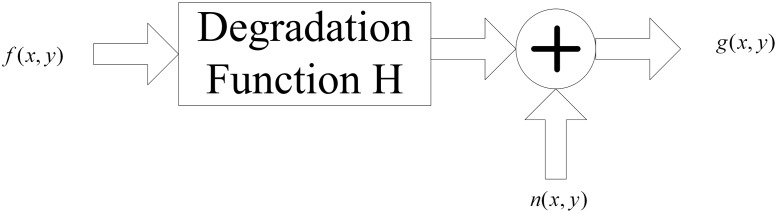
Model of image-blurred degradation.

A motion-blurred image is generated by the relative motion between the target and the camera during the image’s exposure time. The study of motion blur produced by uniform motion is of general significance, because the variable speed and the linear motion blur can be approximately considered as uniform motion in the shooting moment. Following motion-blurred degradation and additive noise superposition, the output result is a blurred image [22]. This degradation process can be shown in Fig 1.

In this model, the output is calculated by means of the following formula [23]

g(x,y)=f(x,y)⊗h(x,y)+n(x,y) (1)

where *g*(*x*,*y*) is the blurred image, *f*(*x*,*y*) is the undegraded image, *n*(*x*,*y*) is system noise, *h*(*x*,*y*) is the point spread function (PSF), and ⨂ is the convolution in spatial domain. Since the space domain convolution is equal to frequency domain multiplication, the frequency domain representation of Eq (1) is *G*(*u*,*v*) = *F*(*u*,*v*)*H*(*u*,*v*)+*N*(*u*,*v*).

Motion-blurred restoration involves reversing the image degradation process and adopting the inverse process to obtain clear images. Motion-blurred is one case that was featured in the model of Lin et al [22]. The model assumes that the target or camera moves at a certain speed and direction, and a distance, *s*, is moved during the exposure time, T. Regardless of the effect of noise, it can be presented by the formula$g(x,y)=\frac{1}{T}\int_{0}^{T}f(x-x(t),y-y(t))dt$. In addition *x*(*t*),*y*(*t*) are the time-varying components of motion in the x-direction and y-direction.

The Fourier transform of *g*(*x*,*y*) is

$$\begin{array}{l} G(u,v)=\int_{\text{-}\infty }^{\infty }\int_{\text{-}\infty }^{\infty }g(x,y)e^{-j2\pi (ux+vy)}dxdy\\ \begin{array}{ccc} & & =\frac{1}{T}\int_{0}^{T}e^{-j2\pi [ux(t)+vy(t)]}dt \end{array}F(u,v) \end{array}$$ (2)

The spectrogram of the blurred image is the modulus square of Eq (2), which leads to an un-degraded image where the phase shift is absorbed, since its value multiplied by its complex conjugate is equal to unity. By defining $H(u,v)=\frac{1}{T}\int_{0}^{T}e^{-j2\pi [ux(t)+vy(t)]}dt$. Eq (2) can be expressed in the form of *G*(*u*,*v*) = F(*u*,*v*)•*H*(*u*,*v*), so it is possible to restore the motion blurred image if *H*(*u*,*v*) is known.

The inverse Fourier transform of *H* is *h*(*x*,*y*) = 1/*vT* = 1/*s*. This shows that, the unknown variable is *s*, which includes the direction and scale. So theoretically, if the two parameters are known, it is possible to obtain the spectrogram and restored image of the blurred image. Therefore, a new method based on the border in the spatial domain is proposed to extract the parameters quickly and efficiently.

From the perspective of image restoration, the minimum mean square error filtering (Wiener Filtering) method is adopted, which can avoid the effect of white noise. The method is shown by the expression

$$\hat{F}(u,v)=[\frac{1}{H(u,v)}\frac{|H(u,v){|}^{2}}{|H(u,v){|}^{2}+K}]G(u,v)$$ (3)

where *K* is a modifying factor representing the power ratio of the noise and the signal. Because this value cannot be accurately obtained, a fixed value is replaced in practice. The inverse Fourier transform of $\hat{F}(u,v)$ is the restored image.

### 2.2 Parameter extraction based on border in the spatial domain

Through the above description about the restoration algorithms of motion-blurred images, it is obvious that extracting the movement direction and scale is a key step in the process, and that determining how to detect the two parameters quickly and accurately is the key problem. There were already some algorithms in existence that can extract these parameters, and most of these algorithms try to do it in the frequency domain by measuring the zero pattern of the blurred image. However, these methods lack of robustness and are easily affected by noise. Therefore, it is necessary to find a new method that can calculate the two parameters.

#### 2.2.1 Border deformation description of motion-blurred traffic signs

**Fig 2:**
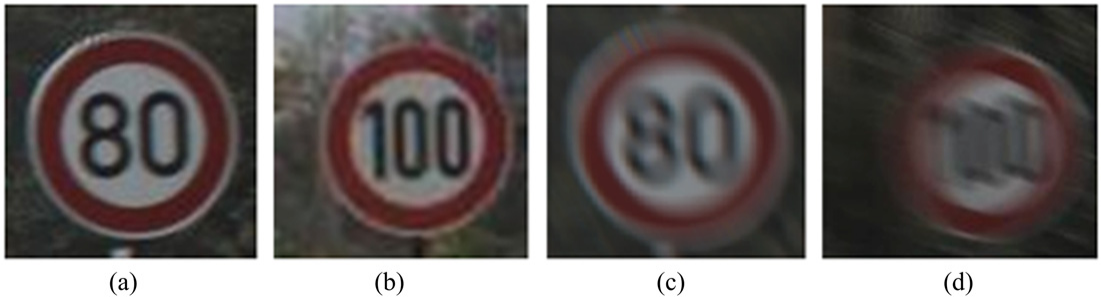
Traffic signs images. (a) and (b) sharp images. (c) and (d) motion-blurred images.

Traffic signs usually have a color circle border and a black number, or some other patterns, within it, as shown in Fig 2(a) and 2(b). By analyzing the traffic signs in motion-blurred images, we found that no matter what the direction of motion is, the width of the border will deform regularly. Fig 2(c) and 2(d) feature the motion-blurred images of Fig 2(a) and 2(b). When comparing the blurred image with the sharp image, we can conclude that the border will become wider on the front and back sides in the direction of motion, and that the blurred image has lower color saturation. On the right and left sides, the boundary change is small compared with the sharp image.

**Fig 3:**
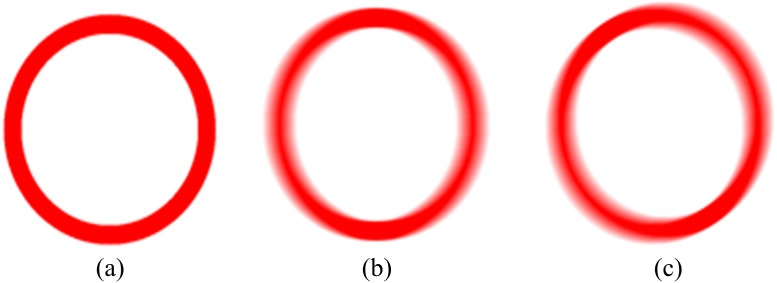
Motion-blurred simulation of traffic sign's border. (a) Original border of the traffic sign. (b) motion-blurred image of the border by 0° and 15 pixels, and (c) motion-blurred image of the border by 30° and 15 pixels.

In order to explain the changing laws of the border clearly, the blurring process is simulated. Fig 3(a) shows the border of the traffic sign. We were then able to produce motion blur in the direction of 0° and 30°, and we were able to extend it by 15 pixels. Lastly, we obtained the border's motion-blurred images [see Fig 3(b) and 3(c)].

It can be clearly observed that motion causes the border's regularity to change. Fig 3(a) is the original border before it became motion blurred. Fig 3(b) moved in a direction of 0°. The border around the 0° direction had apparently become blurred and its boundary became wider. Moreover, its color saturation dropped much more markedly than in the border around the 90° direction. Fig 3(c) is an image that presents similar features, though the direction is 30°.

#### 2.2.2 Parameter extraction model

In the case of horizontal motion [Fig 3(b)], each line of the image is a sequence that could be expressed as *f*(*x*,*y*), and each sequence is considered as a one-dimensional sequence, which can be expressed as *f*(*x*). Assuming that the value of the border pixels is 1, and others are 0..Both *a* and *b* are the boundaries of the traffic sign borders.

The sequence moved *n* pixels towards the right, and n<b-a. Through inverse Fourier transform, it can be known that *h*(*x*) = 1/*n (*0≤*x*≤*n*-1*)*.Ignoring the effect of noise, we can express the sequence after motion as

$$g(x)=\sum_{k=-\infty }^{+\infty }f(k)h(k-x)$$ (4)

By simplifying Eq (4), we can get

$$g(x)=\{\begin{array}{cc} \frac{1}{n}x-\frac{a-1}{n} & a\leq x<a+n\\ 1 & a+n\leq x\leq b\\ -\frac{1}{n}x+\frac{b+1}{n} & b<x<b+n\\ 0 & others \end{array}$$ (5)

**Fig 4:**
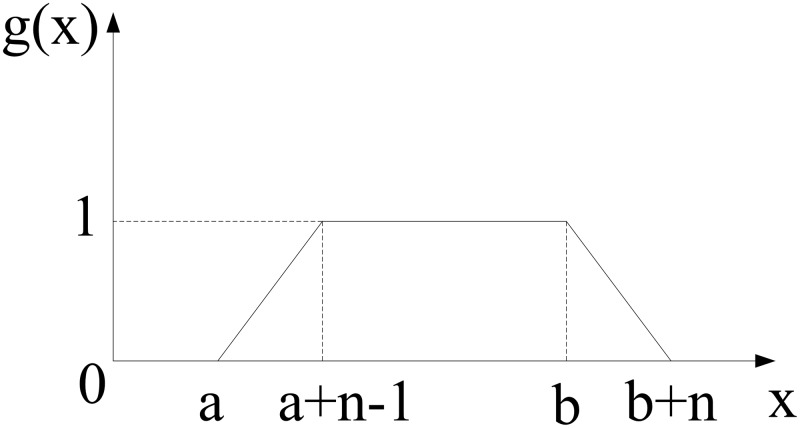
Illustration of the function x solution of Eq 5.

The corresponding illustration of the function x solution of Eq (5) is shown in Fig 4.

From Eq 11 and Fig 4, we can see that when a≤x≤a+n, g(x) grows from 0 to 1. When a+n≤x≤b, the value of g(x) is 1, and when *b*<*x*<*b*+*n*, g(x) decreases from 1 to 0. In the image the saturation of the pixels in the middle of the border is the highest, and it decreases gradually from the middle to the edge of the border. The threshold is set as 1, a+n≤x≤b, and we consider it as the width of the sequence after blurring. This is to say that the width of the pixels whose saturation equals 1 in original sequence is *d*, and the scale of motion is *n*. Thus, the width following motion is *d'* = *d*+*n*.

**Fig 5:**
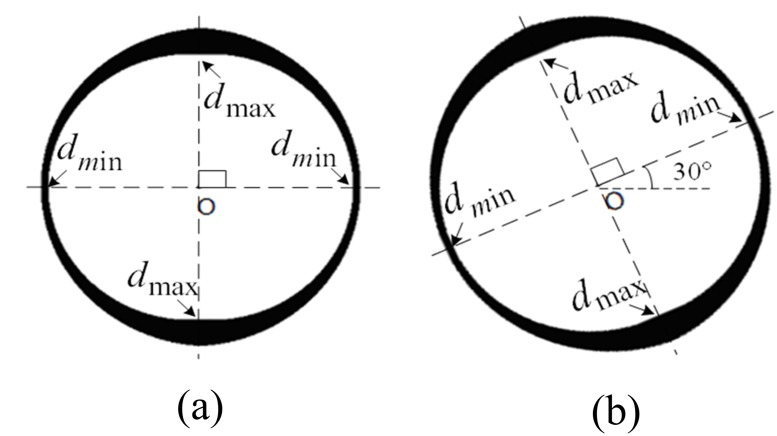
Binary image of motion-blurred border segmented by a certain threshold. (a) Motion-blurred border by 0°, and (b) Motion-blurred border by 30°.

When considering the entire border, the width of the border along the motion direction would apparently change, while the width of the border perpendicular to the motion direction changes little. Finally, the width between the two directions changes gradually, which is shown in Fig 5.

Given that the circle is isotropic, no matter which direction is the image blurred in, the width of the border would change in the same way, which makes it possible to confirm both the blurred direction and scale. Specifically, after measuring the width of all the directions, two maximum values (*d*_max_) and two minimum values (*d*_min_) could be extracted. By connecting two groups of points, respectively, these two lines are perpendicular, and the blurred direction is the direction of the minimum value line.

We can also obtain the scale from the width of the border. Assuming that the width of the border before the motion is d, it is easy to know that the maximum value is *d*_max_ = *d* and that the minimum value is *d*_min_ = *d*-*n*, so the scale is *n* = *d*_max_-*d*_min_. However, the result is easily affected by threshold determination, so the results should be corrected. An appropriate coefficient (*K*) is introduced to the result, so we could obtain the corrected result, n = K *(*d_*max*_-d_*min*_ *)*.

Thus far, the parameters of direction and scale are extracted from the motion-blurred images.

### 2.3 Border deformation detection for image restoration

**Fig 6:**
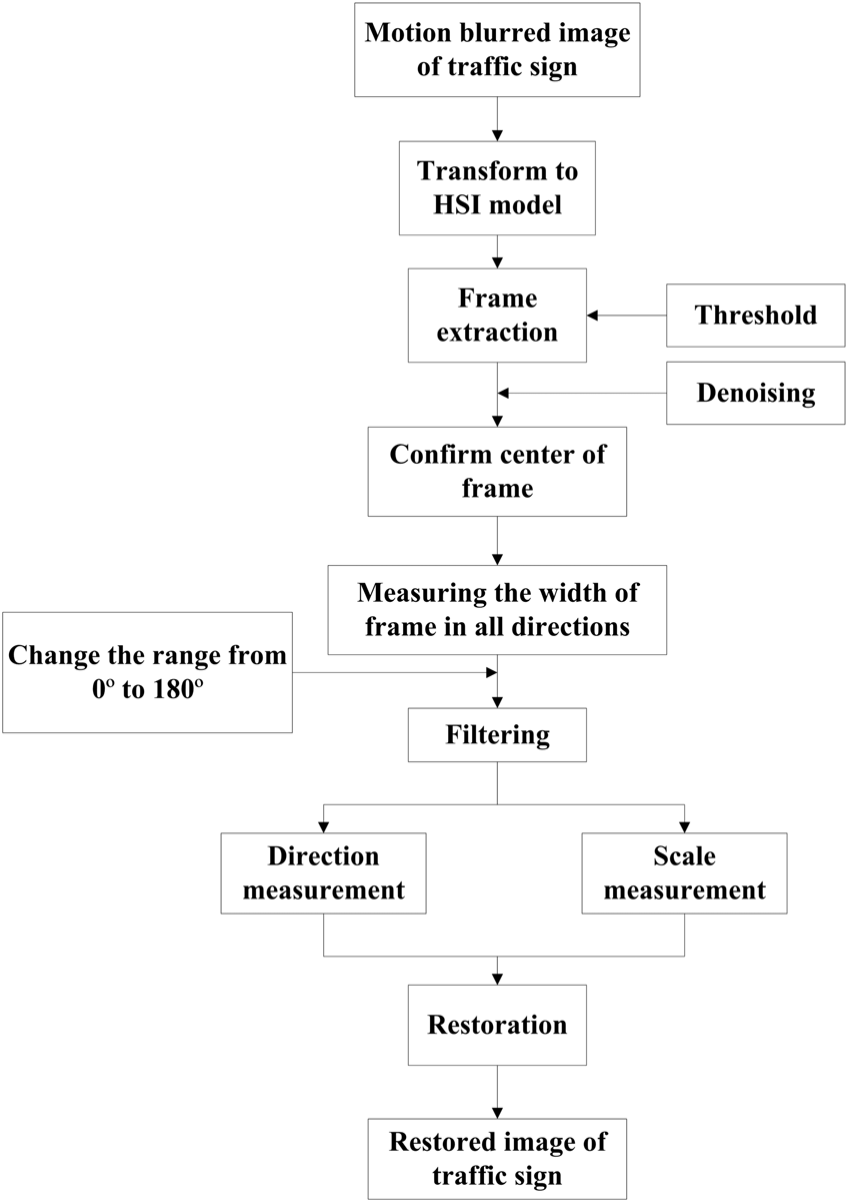
Block diagram of the restoration algorithm.

According to the border deformation characteristics of the motion-blurred images of traffic signs, the process of realizing the algorithm is drawn in Fig 6. The first step in the process is to remodel the image from RGB (Red, Green, Blue) to HIS (Hue, Saturation, Intensity); then, the traffic sign border should be extracted by defining the appropriate threshold of HSI. After setting the center of the border, we can measure the width of the border in all directions. Then, the two parameters of direction and scale can be calculated. At last, the motion-blurred image of the traffic sign can be restored using the results obtained with this method.

#### 2.3.1 Border extraction

There are some methods that can be used in border extraction [24, 25]. Since the border is blurred, the traditional method cannot meet the requirements needed to extract the borders with accuracy and integrity.

**Fig 7:**
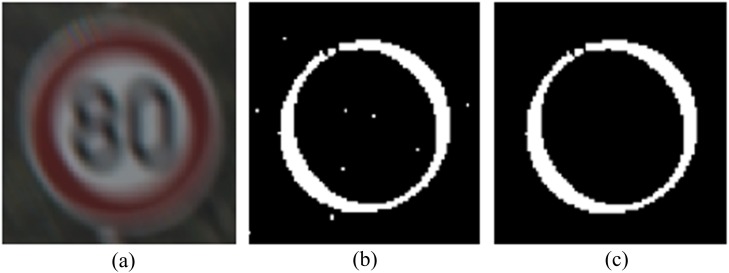
Result of border extraction using the HSI model. (a) Motion-blurred image of traffic sign, (b) result of the border extraction with some noise, and (c) denoised image as a result of border extraction.

The traffic sign's borders are red, so it is possible to confirm whether the pixel belongs to the border or not by checking its color. The RGB image cannot confirm the color directly, so the method is based on the HSI model. The HSI color space is well suited to describe color in a way that is practical for human interpretation [26]. In the HSI model, the variation of light does not greatly affects the value of hue [27], so it is easy to confirm what the color of the pixel is. The border of the traffic sign is red, and according to the statistical results, the *H* values of most border pixels fall in the range of 0° ~36° and 324 ° ~360°. In addition, in this method the intensity component was not used in the calculation, which reduces much computational capacity. There may be some noise in the resulting extraction; as such, we use a filter to remove it. The results of this method are shown in Fig 7.

#### 2.3.2 Measurement of border width in all directions

In order to measure the width of the border in all directions, we should count the pixels from the center of the circle to the edge of the image in all directions, confirming the center of the border is the first step. Theoretically, the blurred border is centrosymmetric, so the center of the border is the center of gravity. Assuming the size of the image is M×N, the center of the border *O*(*O*_*x*_,*O*_*y*_) is represented by $O_{x}=\sum_{n=1}^{N}nf(n)/\sum_{n=1}^{N}f(n)$ and $O_{y}=\sum_{m=1}^{M}mf(m)/\sum_{m=1}^{M}f(m)$. In addition, *f*(*n*) and *f*(*m*) are the summation of the white pixels in the *m* column or in the *n* row.

**Fig 8:**
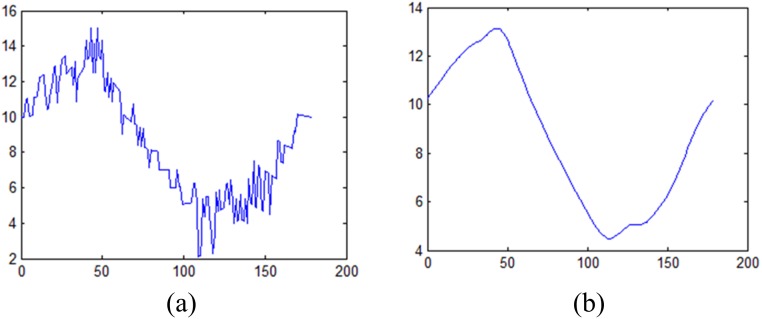
Measurement result of motion-blurred traffic sign. (a) Original measurement result, and (b) result after filtering.

To measure the width of the border, we compute the border by a step of 1°. Through theoretical analysis and experimental verification, we found that the parameters are the same when the motion blurred directions are θ° and (θ+180) °. So, the direction of the motion-blurred image can be normalized from 0° to 180°. The result of the measurement is shown in Fig 8(a). In order to eliminate noise, the result is processed by the mean filter. Fig 8(b) is the result after filtering.

Following this, we can then determine the two extreme points (the maximum point and the minimum point), and the direction of the minimum point is the motion direction. To ensure that the results are more precise, we use the direction of the maximum point to correct any errors.

## Results and Discussion

Two different kinds of experiments were designed to test the two parameters respectively. One was to change the blurred scale while the blurred direction remained invariant, and the other was to change the blurred direction while the blurred scale was invariant. These experiments could indicate the accuracy, robustness, and scope of the application of these methods.

### 3.1 Evaluation criteria of image restoration

#### 3.1.1 Evaluation criteria of parameter extraction

The absolute error *E*_*a*_ is the measured value between the real value *V*_*r*_ and the measured value *V*_*m*_. This can be calculated by the formula *E*_*a*_ = |*V*_*r*_-*V*_*m*_|.Then the relative error of direction *E*_*dr*_ and the relative error of scale *E*_*sr*_ are respectively defined as

$$E_{dr}=\frac{E_{a}}{180}\times 100\text{\% and }E_{sr}=\frac{E_{a}}{V_{r}}\times 100\text{\%}$$ (6)

#### 3.1.2 Quality evaluation of image restoration

(1) Improved assessment of Gray Mean Grads

The purpose of image restoration is to improve the quality of the image. This paper uses the Gray mean grads (GMG) to evaluate the restored image,

$$GMG=\frac{\sum_{i=1}^{M-1}\sum_{j=1}^{N-1}\sqrt{\frac{{\left[g(i+1,j)-g(i,j)\right]}^{2}+{\left[g(i,j+1)-g(i,j)\right]}^{2}}{2}}}{\left(M-1)(N-1\right)}$$ (7)

where *M* and *N*, respectively, refer to the height and width of the image. The larger the value, the sharper the texture, and that leads to improved image quality. In order to better evaluate the image, we first calculate the GMG of the undegraded image (GMG_ui), the blurred image (GMG_bi) and the restored image (GMG_ri) respectively, and we then calculate the ratio of the GMG of the blurred image (GMG_bi) to the GMG of the undegraded image (GMG_ui),

$$GMG_{bu}=\frac{GMG\_bi}{GMG\_ui}\times 100\%$$ (8)

as well as the ratio of the GMG of the restored image (GMG_ri) to the GMG of the undegraded image.

$$GMG_{ru}=\frac{GMG\_ri}{GMG\_ui}\times 100\%$$ (9)

Greater values indicate that the two images are closer.

(2) Laplacian Sum

The Laplacian sum (LS) is a global sum value of the image, and is calculated with the following formula,

$$LS=\frac{\sum_{i=2}^{M-1}\sum_{j=2}^{N-1}\left|\begin{array}{l} 8g(i,j)-g(i,j-1)-g(i-1,j)-g(i+1,j)-g(i,j+1)\\ -g(i-1,j-1)-g(i-1,j+1)-g(i+1,j-1)-g(i+1,j+1) \end{array}\right|}{\left(M-2)(N-2\right)}$$ (10)

When an image is much clearer, its edge is more distinct and the LS value is much greater. The GMG and LS values are the most common No-Reference Quality Assessment for motion blurred image.

(3) Recognition rate

The restored traffic sign images using this algorithm are recognized using a recognition system that has been used to test the effectiveness of the algorithm in this paper.

$$R=\frac{N_{c}}{N_{s}}\times 100\%$$ (11)

where *N*_*s*_ represents the total number of test samples, and *N*_*c*_ is the number of test samples that are recognized correctly.

### 3.2 Validation of parameter extraction

When the blurred direction is fixed and the scale changes from 2 pixels to 18 pixels, the results of the measurements obtained in this method are shown in Table 1.

**Table 1:** Results of measurement when direction is fixed at 30° and scale is variation.

Number	Direction (°)				Scale (pixels)			
	Measured value(*V*_*m*_)	Real value (*V*_*r*_)	Absolute error (*E*_*a*_)	Relative error(%)	Measured value(*V*_*m*_)	Real value (*V*_*r*_)	Absolute error (*E*_*a*_)	Relative error(%)
1	23	30	7	3.89	2.06	2	0.06	3.0
2	29	30	1	0.56	5.07	5	0.07	1.4
3	29	30	1	0.56	7.23	7	0.23	3.3
4	28	30	2	1.11	10.08	10	0.08	0.8
5	29	30	1	0.56	11.48	12	0.52	4.3
6	30	30	0	0	13.25	15	1.75	11.7
7	29	30	1	0.56	15.10	18	2.90	16.1

**Fig 9:**
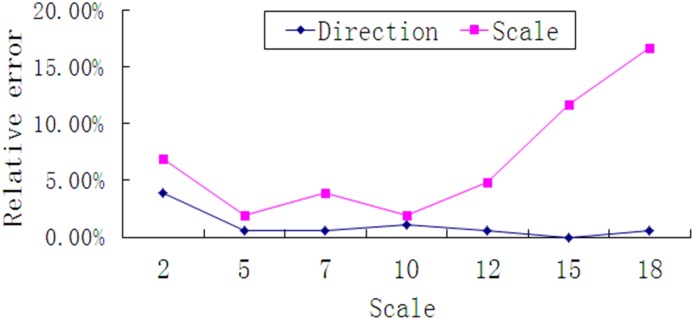
Relative error of scale and direction with fixed direction.

In order to make the results more intuitive, the relative error of the parameters is shown in Fig 9.

From Table 1. and the corresponding curve in Fig 9, it can be known that when direction is invariant, the results of the direction measurement are more precise than the scale measurement; this is because it is hard to confirm the threshold and the coefficient (*K*) at the border extraction step when illumination changes. The accuracy of the direction measurement is low when the scale is less than 5 pixels. However, there is no need to restore the image because the image is clear enough to recognize. The accuracy of the scale measurement is acceptable when the scale is between 5 and 12 pixels. When the scale is higher than 12 pixels, the error is too large to accept. Additional observations show that the traffic sign border's width of in the sharp image is about 10 pixels, so the algorithm is not fit for use in a situation when the blurred scale is much larger than the width of the border.

The blurred scale was also fixed at 10 pixels and the direction was changed from 0° to 180°, the results of this measurement are shown in Table 2.

**Table 2:** Results of measurement when the scale is fixed at 10 pixels and direction is variable.

Number	Direction (°)				Scale (pixels)			
	Measured value(*V*_*m*_)	Real value (*V*_*r*_)	Absolute error (*E*_*a*_)	Relative error(%)	Measured value(*V*_*m*_)	Real value (*V*_*r*_)	Absolute error (*E*_*a*_)	Relative error(%)
1	0	0	0	0	10.40	10	0.4	4.0
2	28	30	2	1.1	10.08	10	0.08	0.8
3	59	60	1	0.6	9.27	10	0.73	7.3
4	88	90	2	1.1	9.52	10	0.48	4.8
5	123	120	3	1.6	10.67	10	0.67	6.7
6	153	150	3	1.67	10.12	10	0.12	1.2
7	178	180	2	1.1	10.43	10	0.43	4.3

**Fig 10:**
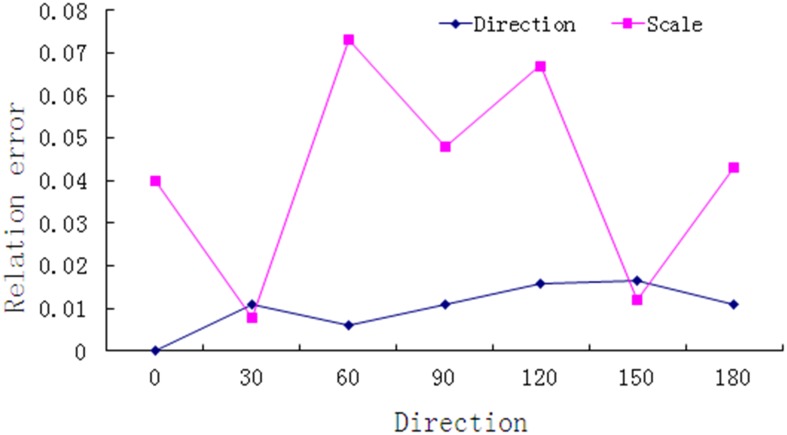
Relative error of scale and direction with fixed scale.

To make this result more intuitive, the relative error of the parameters is shown in Fig 10.

From Table 2. and Fig 10, it can be shown that the results are fairly accurate, the relative error of the direction measurement was below 2%, and the scale measurement was below 8%. Compared with the scale, the directions are measured more accurately and are stable.

### 3.3 Validation of image restoration

**Fig 11:**
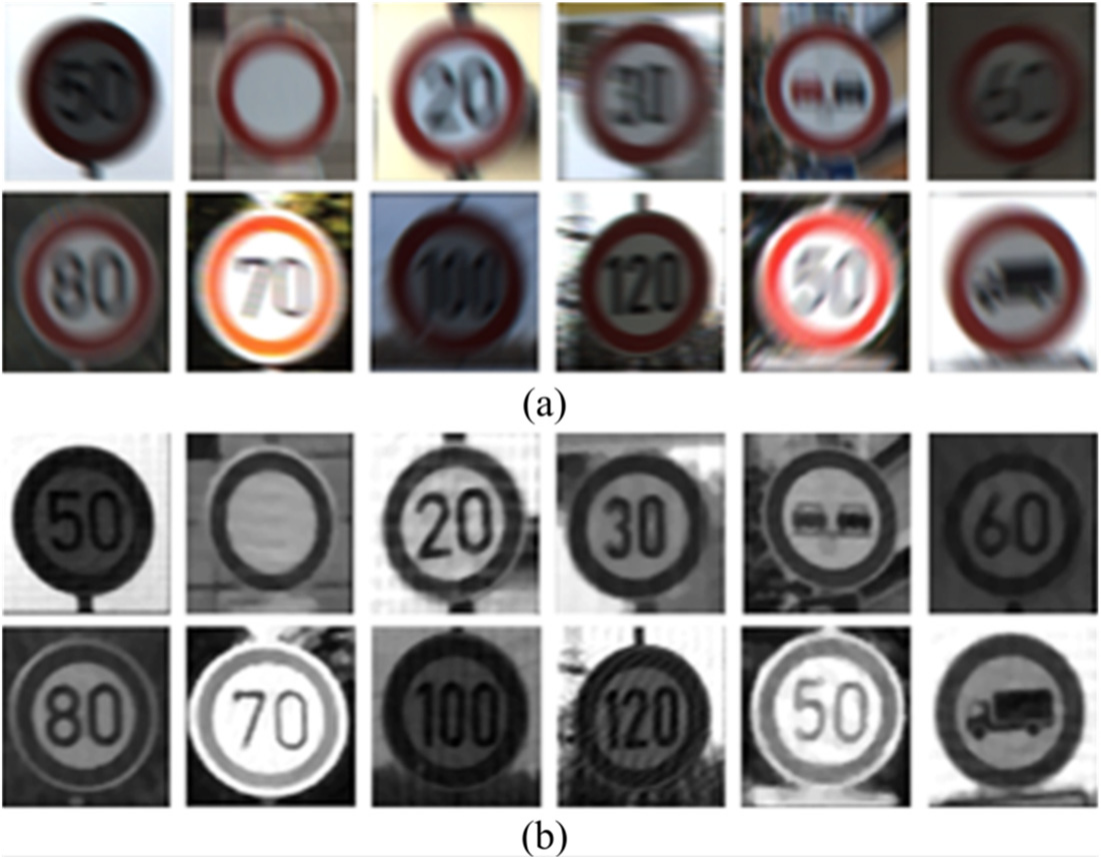
Results of traffic sign restoration by our method. (a) Motion-blurred images of traffic sign, and (b) restored images of traffic sign.

**Fig 12:**
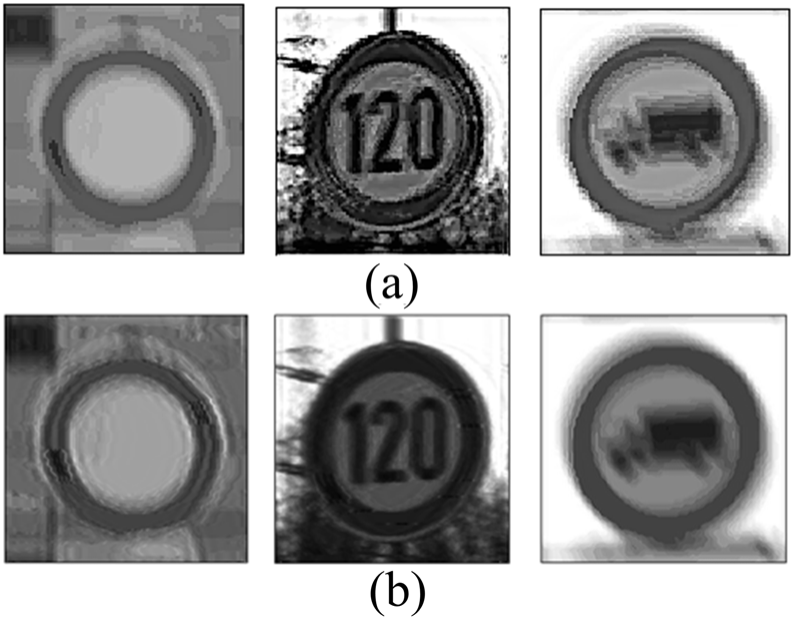
Part of the restoration results of comparison methods. (a) restoration results using method [11], and (b) restoration results using method [13].

Using the parameters measured by this method to restore the motion-blurred image, we obtained good restoration results. Fig 11 shows part of the blurred images and the corresponding restored images. We also compared our proposed method with two commonly used methods of restoration. The first one is based on the blind deconvolution method, which was introduced by Jan Kotera *et al*. in [11], and the second one based on Lucy-Richardson method was introduced by Manoj Kumar Singh *et al*.in [13]. Both of these methods do not require any information about the image, and the iteration was set to around 30 iterations in the experiment. Fig 12 shows part of the restoration results obtained by both method [11] and [13], which can result in slightly better visualization from these blurred images. Fig 12(a) shows the results obtained using the blind deconvolution method [11], and Fig 12(b) shows the results obtained using the Lucy–Richardson method [13].

From the results of the restoration, we can see that the proposed method yields the best restoration results whenever the overall visual effect and local visual details are less noisy and are of higher integrity. As such, our method is efficient in automatic detection system.. Most of these motion-blurred traffic sign images can be restored well, while some of these restorations led to the generation of stripes, as caused by the Wiener Filter. Our method can also be used for motion blurred images featuring different contrast levels.

In Fig 12, the method proposed by Jan Kotera *et al*. and the method proposed by Manoj Kumar Singh *et al*. both led to the problems with noise amplification and ringing effects, and the details of texture and edge were not clear enough for subsequent processing. In addition, they did not work particularly well for motion blurring images, because most the test data cannot be deblurred in these experiments.

**Fig 13:**
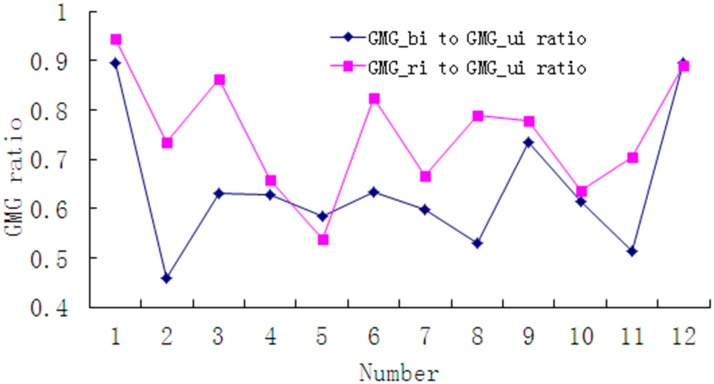
GMG ratio curves of part images.

Some images were randomly selected from a total of 217 samples and their GMG ratio was subsequently calculated, and the results are shown in Fig 13. The texture information following restoration was worse when compared to that of the blurred image in each individual image, because the direction and scale differences led to errors in parameter extraction. However, it can be determined that most of the traffic sign image restorations can improve the image quality, as it features, clearer texture. This shows that the algorithm is both stable and reliable. Table 3 shows the LS values of the motion blurred images and the restored results in Fig 12. In general, the proposed method obtained the best visual effect and featured the highest values when meeting the assessment criteria, thus, its restoration result is better than that of the blind deconvolution and Lucy-Richardson methods.

**Table 3:** Comparison of LS values.

	Motion Blurred image	Blind Deconvolution method	Lucy-Richardson method	Proposed method
Img.1	23.01	25.62	28.05	29.18
Img.2	24.45	28.76	25.14	26.83
Img.3	23.20	26.24	30.40	32.70
Average	23.55	26.51	27.86	29.57

The traffic sign images restored by this algorithm are recognized using the recognition system based on the SVM, which is employed to test the effectiveness of the algorithm. There are 217 samples in all, and the recognition rates of the motion-blurred traffic sign images and their corresponding restored images are shown in Table 4. The results of the image recognition show that the motion-blurred traffic signs are hard to recognize, there were only 22 traffic signs that were recognized in a total of 217 images, and the recognition rate was low at 10.14%. However, following restoration, 191 traffic signs can be recognized, and the recognition rate subsequently increases to 88.02%. In real data featuring traffic signs, about 10% images are blurred by motion. Using this method, the recognition rate can be improved to about 77.84%. This demonstrates that the restoration algorithm is effective, and that the restoration of motion-blurred images is necessary in image detection.

**Table 4:** Comparison of recognition rate results.

	Recognized correctly	Recognized incorrectly	Recognition rate (%)
Motion-blurred traffic images	22	195	10.14
Restored traffic images using border deformation detection	191	26	88.02

## Conclusions

This paper proposed a new method to measure two important parameters—direction and scale—of motion-blurred traffic signs in the spatial domain. Through our experiments, it was determined that the results are accurate and that the error rates are acceptable. This method is robust, and it can reduce the impact of changing illumination on parameter extraction. Using the measured parameters to restore the motion-blurred traffic sign images, we obtained good results that could meet the system's requirements in image recognition. The results illustrated that the method can deal with recognition-based problems associated with motion-blurred traffic sign images. Compared with the methods based on the frequency domain, the impact of noise on parameters extraction is much smaller. In conclusion, application of the algorithm offers an advantage in traffic signs recognition. This method can improve the performance of the DAS and help to improve automatic driving and road safety.

As for future work, we will continue to investigate this subject by providing a more detailed background of this problem, and we will work to improve the robustness of border extraction with more suitable features in reducing the effects of the environment.
